# The Classification of Axial Deformity in Patients with Basilar Invagination

**DOI:** 10.1111/os.13487

**Published:** 2022-10-12

**Authors:** Qiting He, Jiankang Cao, Huichao Tian, Bin Chen, Xincheng Fan, Shaoyi Wang, Yunpeng Zhao, Jianlu Wei, Lin Nie, Xin Pan, Lei Cheng

**Affiliations:** ^1^ Department of Orthopedic Surgery, Qilu Hospital of Shandong University, Cheeloo College of Medicine Shandong University Jinan China; ^2^ Department of Pain, Qilu Hospital of Shandong University, Cheeloo College of Medicine Shandong University Jinan China; ^3^ Department of Orthopedic Surgery Liaocheng People's Hospital Liaocheng China; ^4^ Department of Orthopedic Surgery Taian City Central Hospital Taian China

**Keywords:** axis, axis deformity, basilar invagination, dysplasia

## Abstract

**Objective:**

To summarize the variation types of the axis in patients with basilar invagination (BI), then propose a classification scheme of the axis deformity.

**Methods:**

From December 2013 to September 2020, 92 patients (male 42, female 50) who were diagnosed with BI were studied retrospectively. Based on the imaging data of CT, the width and height of the axis pedicle and the sagittal diameter of the lateral mass were measured in each patient. According to the development of axis pedicle and lateral mass, the types of axis variation were summarized, and then the classification scheme of axis deformity was put forward.

**Results:**

All cases were analyzed and axis deformities were divided into four types. Type I: the axis is basically normal (53 cases, 57.6%). Type II: axis lateral mass is dysplasia (eight cases, 8.7%), which includes two subtypes: type IIA, the axis unilateral lateral mass is dysplasia (three cases); type IIB, the axis bilateral lateral masses are all dysplasia (five cases). Type III: axis pedicle is dysplasia (11 cases, 12%), which is subdivided into two subtypes: type IIIA, the axis unilateral pedicle is dysplasia (six cases); type IIIB, the axis bilateral pedicles are all dysplasia (five cases). Type IV: axis pedicle and lateral mass are all dysplasia (20 cases, 21.7%), this type contains the following four subtypes: type IVA, the unilateral axis pedicle and unilateral lateral mass (contralateral or ipsilateral) are all hypoplasia (four cases); type IVB, the unilateral axis pedicle and bilateral lateral masses are all hypoplasia (five cases); type IVC, the bilateral axis pedicles and unilateral lateral mass are all dysplasia (seven cases); type IVD, the bilateral axis pedicles and bilateral lateral masses are all dysplasia (four cases). The left and right abnormal lateral mass sagittal diameter (Type II) was (7.23 ± 1.39) mm and (5.96 ± 1.37) mm, respectively, the left and right abnormal pedicle width (Type III) was (2.61 ± 1.01) mm and (3.23 ± 0.66) mm, respectively, left and right abnormal pedicle height (Type III) was (5.43 ± 2.19) mm and (4.92 ± 1.76) mm, respectively. Moreover, the classification scheme has good repeatability and credibility.

**Conclusions:**

The classification about axis deformity could provide personalized guidance for axis screw placement in the BI and other upper cervical surgery, and axis screw placement errors would be effectively avoided.

## Graphical abstract

Axis deformities were divided into four types. Type I: the axis is basically normal. Type II: axis lateral mass is dysplasia. Type III: axis pedicle is dysplasia. Type IV: axis pedicle and lateral mass are all dysplasia. The axis variation rate of patients with basilar invagination was as high as 42.4%, of which type IV was the most common.

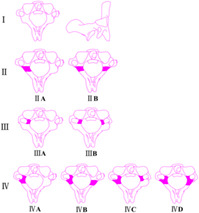

## Introduction

Basilar invagination (BI) is a compression syndrome of neuro spinal cord, which results from the complex bone structure deformity of the cranial cervical junction, the tissues of skull bottom around the foramen magnum and the odontoid process moves upward into the cranial cavity. It's reported that BI is more prevalent in India, Brazil, and other countries, it usually occurs in 10–30‐year‐old patients, but also older patients, and 25%–35% of patients have neurological abnormalities^1^. The displacement of the axis odontoid results in the stenosis of the foramen magnum and the compression of brainstem, ventral cervical spinal cord, cerebellum, and peripheral vascular, which in turn cause a series of clinical symptoms. The main symptoms are neck pain, limb weakness, sensory numbness, and other neurological symptoms^2^, ^3^. Its pathogenesis is mostly related to the flat skull bottom, occipital cervical fusion, and Kleip–Feil malformation, which result from congenital embryonic abnormality, and may also be related to the acquired osteomalacia around the skull bottom, such as rheumatoid arthritis, Paget's disease (malformed osteitis), hyperthyroidism, rickets, infection, and tumor can also be related to compensatory mechanism of atlantoaxial instability^4^. The main imaging manifestations are as follows, atlantoaxial dislocation, odontoid process backward and upward into the occipital foramen, compression of brainstem or medulla oblongata.

At present, the diagnosis of BI mainly depends on imaging diagnosis. In 1939, Chamberlain^5^ first reported the classical Chamberlain line through a large number of imaging studies. McGregor^6^ reported the Gregor line in 1948. With the advent of CT and MRI, more and more imaging parameters for the diagnosis of BI was studied, such as McRae line, Wackenheim clival line, double mastoid line, digastric sulcus line, atlantooccipital joint angle, basal angle, and so on. These imaging parameters make it easier, more accurate, and more specific in the diagnosis of BI. The majority of patients with BI need surgical treatment, and with the improvement of reduction technique and internal fixation, the posterior occipital cervical fusion surgery is an effective method for the treatment of BI and other various craniocervical junction diseases^7^, ^8^. In 1994, Goel first reported the posterior atlantoaxial screw‐rod (plate) fixation technique, many scholars improved this technique, using it widely in posterior occipital cervical fusion surgery. It gradually became the main internal fixation system^9^. Some studies reported that the fusion rate could reach 91.8% in the posterior occipital cervical fusion surgery^10^. When posterior occipital cervical fusion surgery was performed in the BI patients, axis fixation occupied a very important position in fusion surgery. However, the surgical risk of BI is very high, and due to the various variations of axis, it is easy to damage the vertebral artery, puncture the inner wall of the pedicle, and damage the spinal cord in the process of screw placement, so axis screw placement is more difficult and important. Moon^11^ reported that 13 of 48 patients (27.1%) with atlantoaxial instability due to congenital skeletal anomaly had a high‐riding axis vertebral artery and 18 of 52 patients (34.6%) had acquired atlantoaxial instability. Many scholars have studied the normal anatomical structure of the axis, mainly on the pedicle of the axis, such as pedicle height and pedicle superior angle^12^. Although these findings shed light on the normal axis structure and facilitating screw placement in patients without axis malformation, there are many risks associated with intraoperative screw placement in patients with axis variation. However, there are no detailed studies on axis malformation in BI patients, so we need to study the axis variations to reduce the risk of screw placement of the axis.

We found that there are varying degrees of variation in the axis pedicle and lateral mass of most patients with BI by studying the CT, and these variations would have some great impacts on the choice of axis screw placement scheme. At present, there are many reports of axis pedicle deformities, but the classification of axis deformities in patients with BI has not been clearly reported. Therefore, the purpose of our research was to analyze and classify the axis deformities of BI patients, and propose a classification scheme of the axis deformity in patients with BI, then provide personalized guidance for axis screw placement in the BI and other upper cervical surgery by the classification scheme.

## Materials and Methods

### Inclusion and Exclusion Criteria

Inclusion criteria: (i) the selected patients were diagnosed with BI; (ii) the patients had complete cervical CT imaging data. Exclusion criteria: (i) atlantoaxial fracture; (ii) atlantoaxial tumors; (iii) atlantoaxial infection; (iv) previous upper cervical spine surgery.

### Imaging Indicators of BI

We used Chamberlain line as the diagnostic criterion of BI. A line is drawn from the posterior edge of the hard palate to the opisthion at standard cervical lateral radiograph or sagittal reconstruction of CT scan. If the axis odontoid tip exceeded this line by 3.5 mm it was diagnosed with BI.

We reviewed and analyzed the imaging data of 92 patients with BI who were treated in the department of spinal surgery, Qilu Hospital of Shandong University, from December 2013 to September 2020, the research proposal was approved by the Shandong University Qilu Hospital Ethics Committee (approval number: 2019264). The four trained radiologists mainly read the cervical CT images of each patient, the CT (GE, Optima CT660, 128 T) scan thickness was 0.3 mm. We measured the pedicle width, pedicle height, and sagittal diameter of lateral mass. IMPAX (version 6.5.5.3020, USA) software was chosen for the measurement.

### Measuring Method

#### Measurement of Axis Pedicle Width

**FIGURE 1:**
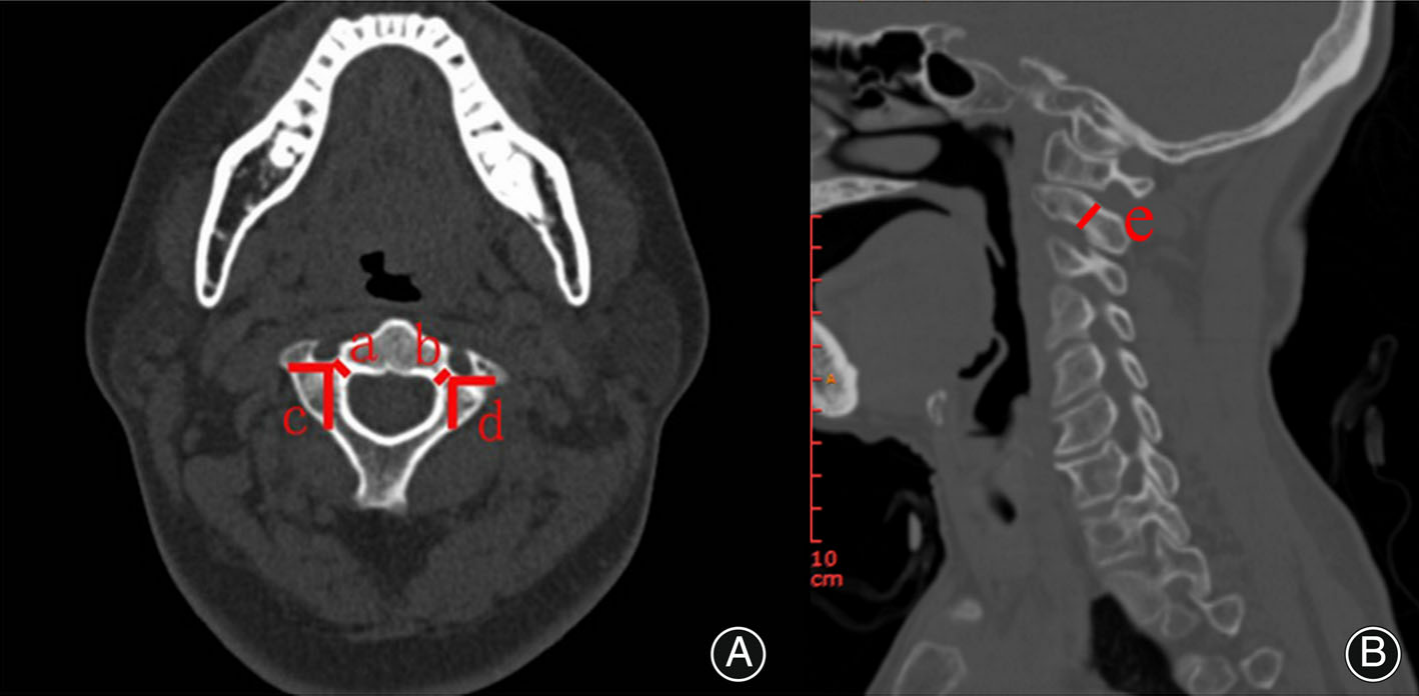
The measurement method of axis pedicle width, pedicle height, and lateral mass sagittal diameter. (A) The measurement method of axis pedicle width (a, b) and lateral mass sagittal diameter (c, d). (B) The measurement method of axis pedicle height (e)

In the middle transverse layer of the pedicle, we measured the minimum vertical distance from the medial margin of the vertebral artery foramen to the inner edge of the pedicle as axis pedicle width (Figure 1(A)).

#### Measurement of the Sagittal Diameter of the Lateral Mass

Firstly, we made a horizontal line from the posterior edge of the vertebral artery foramen, and then made a vertical line of the horizontal line from screw placement point of axis lateral mass, and finally, we measured the distance from the intersection of the two lines to the screw placement point of axis lateral mass, which was the sagittal diameter of the lateral mass (Figure 1(A)).

#### Measurement of Axis Pedicle Height

In the middle sagittal plane of the pedicle, pedicle height was measured from superior surface of the pedicle to the surface of the vertebral artery foramen (Figure 1(B)).

### Surgical Procedure

Conservative treatment and surgical treatment were selected according to the surgical indications of BI, the surgical indication is neurological symptoms with progressive exacerbation. Fifty patients accepted surgical treatment, 33 patients accepted conservative treatment, and nine patients gave up treatment. The written informed consents were signed by patients who accepted surgery. After the patient was anesthetized successfully, they took the prone position with continuous skull traction (5–10 kg). The posterior median incision of about 7 cm was taken to expose the occipital bone and C_1_–C_4_, then the bilateral cervical muscle was dissected under the periosteum of the cervical spinous process with a periosteal exfoliator until the outer edge of the cervical lamina and lateral mass were exposed. The partial bone around the foramen magnum was removed by bone rongeur to decompress the foramen magnum fully, and then the surgery of occipital cervical fusion and bone graft are performed.

### Analysis of Credibility and Repeatability of Classification System

The classification results of four radiologists were collected to analyze credibility of inter‐observer. Ten days later, four radiologists randomly selected CT scan of patients for further classification again, and the results were collected for repeatability analysis. The credibility and repeatability of classification system were analyzed by Kappa consistency test.

### Statistics and Analysis

SPSS 21.0 statistical analysis software (IBM, USA) was used for data analysis. The data of pedicle width, height, and sagittal diameter of lateral mass were presented as mean ± square deviation, and the statistical difference between the two groups was compared by an independent sample *t*‐test, *p* < 0.05 was statistically significant. The credibility and repeatability of classification system were analyzed by Cohen's Kappa consistency test. Kappa value between 0.75 and 1 indicated very good consistency, Kappa value between 0.4 and 0.75 indicated moderate consistency, Kappa value between 0 and 0.4 indicated poor consistency.

## Results

### The Classification System of Axis Deformity

**FIGURE 2:**
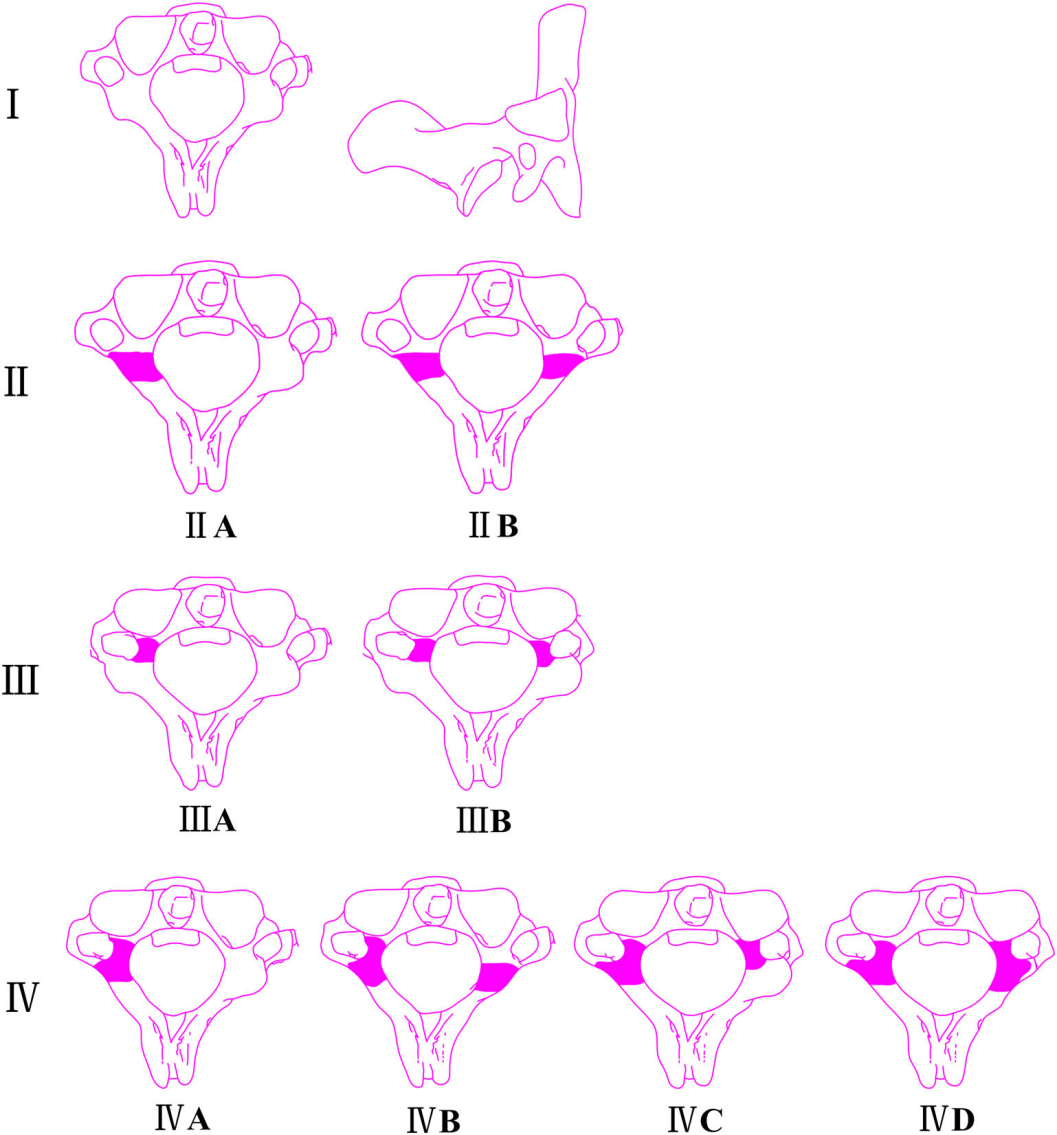
The classification system pattern diagram of axis deformity

**FIGURE 3:**
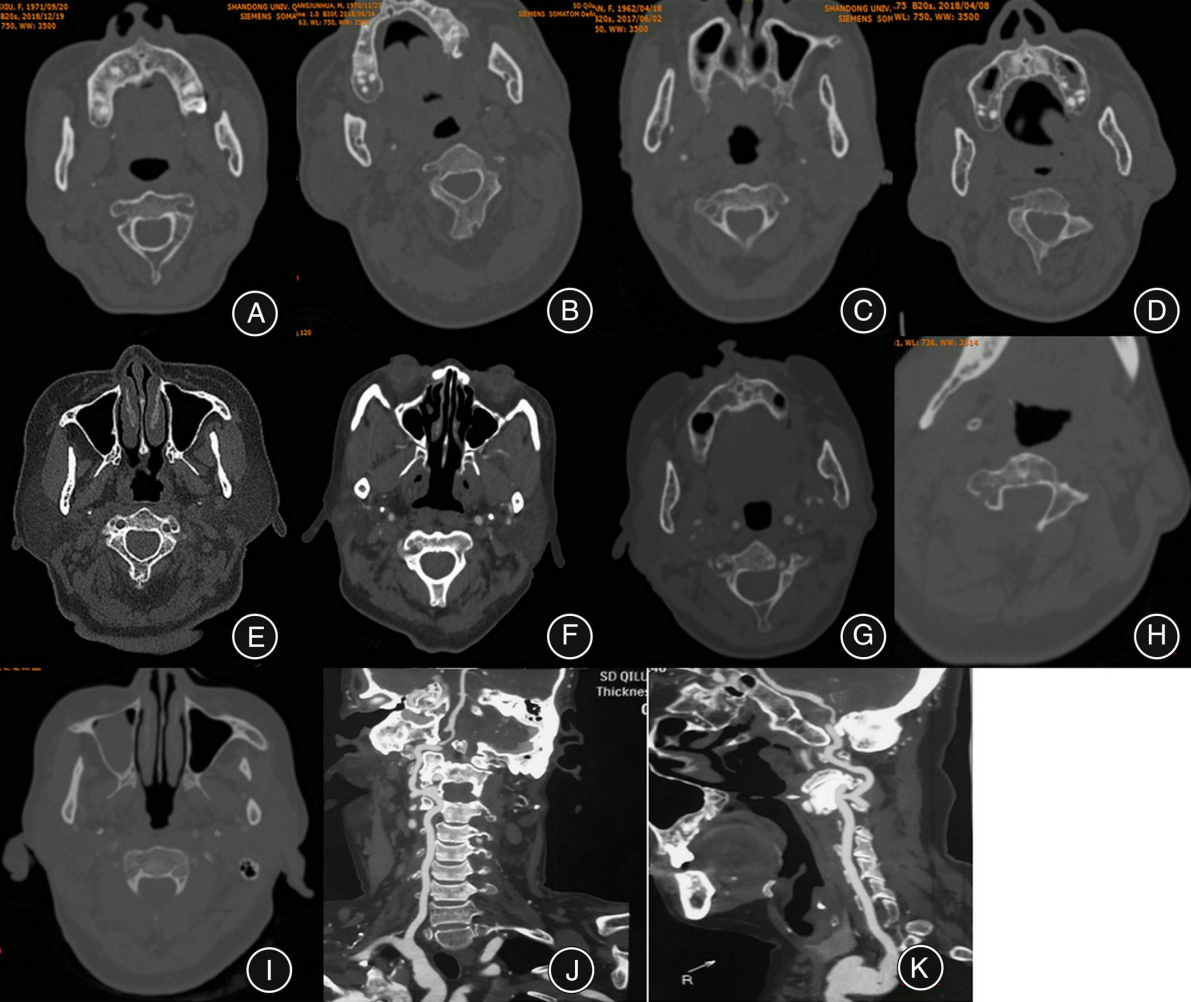
The CT scan of cervical spine in basilar invagination patients. (A) Type I, the axis lateral mass and pedicle are normal. (B) Type IIA, the axis right lateral mass is dysplasia. (C) Type IIB, the axis bilateral lateral masses are all dysplasia. (D) Type IIIA, the axis left pedicle is dysplasia. (E) Type IIIB, the axis bilateral pedicles are all dysplasia. (F) Type IVA, the axis left lateral mass and right pedicle are all dysplasia. (G) Type IVB, the axis bilateral lateral masses and left pedicle are all dysplasia. (H) Type IVC, the axis bilateral pedicles and right lateral mass are all dysplasia. (I) Type IVD, the bilateral axis pedicle and bilateral lateral masses are all dysplasia. (J, K) The coronal and sagittal plane of the high span of the axis vertebral artery.

We divided the axis deformity into four types (Table 1). The pattern diagram of classification system of axis deformity is showed in Figure 2, and Figure 3 is the cervical spine CT of BI patients.

**TABLE 1:** The classification system of axis deformity

Type I: The axis is basically normal	
Type II: The axis lateral mass is dysplasia	Type IIA, the axis unilateral lateral mass is dysplasia Type IIB, the axis bilateral lateral masses are all dysplasia
Type III: Axis pedicle is dysplasia	Type IIIA, the axis unilateral pedicle is dysplasia Type IIIB, the axis bilateral pedicles are all dysplasia
Type IV: The axis pedicle and lateral mass are all hypoplasia	Type IVA, the unilateral pedicle and unilateral lateral mass (contralateral or ipsilateral) are all hypoplasia Type IVB, the unilateral pedicle and bilateral lateral masses are all dysplasia Type IVC, the bilateral pedicles and unilateral lateral mass are all dysplasia Type IVD, the bilateral pedicles and bilateral lateral masses are all dysplasia

Type I: The axis is basically normal.
Type II: The axis lateral mass is dysplasia (the sagittal diameter of lateral mass <9 mm). There are two subtypes: type IIA, the axis unilateral lateral mass is dysplasia; type IIB, the axis bilateral lateral masses are all dysplasia.
Type III: Axis pedicle is dysplasia (the diameter of axis pedicle <5 mm). This type can be subdivided: type IIIA, the axis unilateral pedicle is dysplasia; type IIIB, the axis bilateral pedicles are all dysplasia.
Type IV: The axis pedicle and lateral mass are all hypoplasia and is a combination of type II and type III. This type contains the following four subtypes. Type IVA, the unilateral axis pedicle and unilateral lateral mass (contralateral or ipsilateral) are all hypoplasia. Type IVB, the unilateral axis pedicle and bilateral lateral masses are all hypoplasia. Type IVC, the bilateral axis pedicles and unilateral lateral mass are all dysplasia. Type IVD, the bilateral axis pedicles and bilateral lateral masses are all dysplasia.

### Demographic Data

According to our classification of BI, 92 patients were divided into four types. Forty‐two of the cases were male, average age was 47.3 ± 1.9 years (age range from 30 to 59); 50 cases were female, average age was 46.5 ± 2.5 years (age range from 25 to 69).

Type I, 53 cases (57.6%). Type II, eight cases (8.7%), type IIA (three cases), type IIB (five cases). Type III, 11 cases (12%), type IIIA (six cases), type IIIB (five cases).Type IV, 20 cases (21.7%), type IVA (four cases), type IVB (five cases), type IVC (seven cases), type IVD (four cases).

### The Pedicle Width, Height, and Lateral Mass Sagittal Diameter

The pedicle and lateral mass of type I were basically normal, left and right pedicle width was (6.41 ± 0.72) mm and (6.84 ± 0.80) mm, respectively, the difference was not statistically significant (*p* > 0.05); the left and right lateral mass sagittal diameter was (12.25 ± 1.31) mm and (12.81 ± 1.27) mm, respectively, the difference was not statistically significant (*p* > 0.05); the left and right pedicle height was (7.65 ± 1.02) mm and (8.01 ± 1.2) mm, respectively, the difference was not statistically significant (*p* > 0.05).

For type II, patients had only lateral mass malformation, the left and right abnormal lateral mass sagittal diameter was (7.23 ± 1.39) mm and (5.96 ± 1.37) mm, respectively, but pedicle width and height were normal.

For type III and IV, narrowing of pedicle width was accompanied by undersized height in pedicle height, but the pedicle height was also normal on the side of normal pedicle width. The left and right abnormal pedicle width (Type III) was (2.61 ± 1.01) mm and (3.23 ± 0.66) mm, respectively, left and right pedicle height (Type III) was (5.43 ± 2.19) mm and (4.92 ± 1.76) mm, respectively. For type IV, the left and right abnormal pedicle width was (2.65 ± 0.81) mm and (2.83 ± 1.01) mm, respectively, left and right dysplastic lateral mass sagittal diameter was (6.27 ± 1.32) mm and (6.7 ± 0.95) mm, respectively, left and right pedicle height was (5.04 ± 1.81) mm and (4.52 ± 1.51) mm, respectively. The detailed data of each type is shown in Tables 2 and 3.

**TABLE 2:** The general demographic information for axis deformity classification

Classification	Case	Proportion (%)
I	53	57.6
II	8	8.7
IIA	3	
IIB	5	
III	11	12
IIIA	6	
IIIB	5	
IV	20	21.7
IVA	4	
IVB	5	
IVC	7	
IVD	4	
Total	92	100

**TABLE 3:** The average axis pedicle width, heigh and lateral mass sagittal diameter

Type	*a* (mm)	*b* (mm)	*c* (mm)	*d* (mm)	*e* (mm)	*f* (mm)
I	6.41 ± 0.72	6.84 ± 0.80	12.25 ± 1.31	12.81 ± 1.27	7.65 ± 1.02	8.01 ± 1.2
II	6.50 ± 0.92	6.71 ± 0.72	7.23 ± 1.39	5.96 ± 1.37	8.08 ± 1.32	7.94 ± 1.32
III	2.61 ± 1.01	3.23 ± 0.66	12.65 ± 0.81	12.34 ± 1.33	5.43 ± 2.19	4.92 ± 1.76
IV	2.65 ± 0.81	2.83 ± 1.01	6.27 ± 1.32	6.7 ± 0.95	5.04 ± 1.81	4.52 ± 1.51

*Note*: *a* (mm): the left pedicle width; *b* (mm): the right pedicle width; *c* (mm): the left lateral mass sagittal diameter; *d* (mm): the right lateral mass sagittal diameter; *e* (mm): the left pedicle height; *f* (mm): the right pedicle height. (The data of type II (*c*, *d*), III (*a*, *b*), IV (*a*, *b*, *c*, *d*) were only the diameter of the dysplastic pedicle and lateral mass).

### The Repeatability and Credibility Analysis of Classification System

According to the twice classification results of four radiologists, the average repetition rate of type I, II, III, and IV was 85.2%, 82.425%, 84.15%, 80.5%, respectively, and the mean Kappa value was 0.85, 0.829, 0.849, 0.828, respectively. Therefore, the repeatability of twice classification results performed by any one doctor was very good. Moreover, the average repetition rate of type I, II, III, and IV performed between any two doctors was 82.25%, 79.43%, 80.1%, 77.9%, respectively, and the mean Kappa value was 0.84, 0.821, 0.806, 0.811, respectively, so it indicated this classification had good credibility. Detailed data is shown in Appendix.

## Discussion

### Main Findings of the Study

BI is abnormality at the craniovertebral junction, which is caused by congenital development abnormality or acquired abnormality, of which congenital development abnormality is the main pathogenesis of BI^13^. In our study, we found that there is great difference in the axis of each patient with BI, such as dysplastic lateral mass, narrow pedicle, and high span of vertebral artery, these differences would affect the scheme choice of axis screw placement. Although there are some variations in atlas and other vertebrae, they do not have much effect on screw placement. According to the CT scan of the axis, the axis deformity is divided into four types, the axis variation rate of patients with BI is as high as 42.4%, of which type IV is most common. Moreover, we found the normal left and right pedicle width (type I) are (6.41 ± 0.72) mm and (6.84 ± 0.80) mm, the sagittal diameter of left and right lateral mass are (12.25 ± 1.31) mm and (12.81 ± 1.27) mm, the left and right pedicle height are (7.65 1.02) mm and (8.01 ± 1.2) mm. In our research, we found that the pedicle was narrow when axis pedicle width was less than 5 mm, and the axis lateral mass was dysplastic when the sagittal diameter of lateral mass was less than 9 mm. It may cause surgical risk for the placement of screw when the pedicle was narrow and lateral mass was dysplastic.

#### BI Patients Have a High Rate of Axis Malformation

With the development of occipital cervical deformity, the symptoms will gradually be aggravated and even lead to death^14^. The patients with BI who have neurological symptoms need surgical treatment to relieve spinal cord compression and restore the stability of the craniovertebral junction to prevent the deterioration of the disease^15^. However, there are various variations in axis, so axis screw placement is more difficult^16^. Mandel *et al*.^17^ believed that the minimum width of the middle plane of the axis pedicle should be larger than 5 mm, so that it is safe to insert the 3.5‐mm pedicle screw, otherwise there is a risk for the vertebral artery injury. Naderi *et al*.^12^ measured 160 normal axis pedicles, reported that the width of pedicle on the right side was (6.0 ± 1.5) mm, on the left side was (5.5 ± 1.3) mm. Yamazaki *et al*.^18^ reported there were many variations in vertebral artery at craniovertebral junction, in 10% cases, there were abnormalities in the extraosseous course of vertebral artery at craniovertebral junction, 31% cases had high span of the axis vertebral artery, which seriously affected the axis screw placement during occipital cervical fusion. Vaněk considered it as a high‐riding axis vertebral artery if the axis pedicle width was less than 4 mm or the axis internal height was less than 2 mm, 24.1% of patients had high‐riding axis vertebral artery and bilateral high‐riding vertebral artery was found in 6% of patients^19^.

#### The Classification System of Axis Deformities Has Some Guiding Significance for BI Surgery

There are a variety of surgical schemes for the treatment of BI, through oropharyngeal approach release and reduction combined with posterior fusion fixation^20^, endoscopic odontoidectomy combined with posterior reduction and fixation^21^, transoropharyngeal approach reduction combined with plate internal fixation^22^, only posterior approach to reduction, screw, and rod system fusion and internal fixation^23^, and other surgical schemes. In 2004, Goel^24^ first reported that the treatment of BI with irreducible atlantoaxial dislocations by atlantoaxial joint distraction reduction and fixation through posterior approach. On the basis of Goel, many improved techniques of posterior reduction, fusion and internal fixation were reported^9^, ^25^, ^26^. In recent years, the surgical technique of posterior decompression, reduction, fusion, and internal fixation for the treatment of BI is the main surgical method.

Axis pedicle screw is the first choice in posterior occipital cervical fusion or atlantoaxial fusion, but in our study, we found that there are many axis variations in BI patients, and not every patient is suitable for axis pedicle or lateral mass screw placement. It is necessary to read the imaging data carefully to exclude the axis variation, and to make a personalized screw placement scheme to avoid the risks of axis screw misplacement. We summarized some axis variations, according to our classification, and some risks of axis screw placement could be effectively avoided. This classification scheme can provide some guidance for posterior axis screw placement surgery. According to our surgical experience of BI, we found that the axis pedicle and lateral mass of type I patients are normal, we could choose axis pedicle screw placement or lateral mass screw placement. For type II patients, the pedicle screw was safe, but the lateral mass was dysplasia, the left and right abnormal lateral mass sagittal diameter was (7.23 ± 1.39) mm and (5.96 ± 1.37) mm, respectively, so the lateral mass screw should be avoided. For type IIIA patients with unilateral pedicle dysplasia, we could perform either unilateral pedicle screw to be placed in normal pedicle combine with contralateral mass screw placement or bilateral lateral mass screws placement; for type IIIB patients with bilateral pedicles dysplasia, lateral mass screw placement was feasible, the left and right abnormal pedicle width was (2.61 ± 1.01) mm and (3.23  0.66) mm, respectively, and we should avoid pedicle screw placement. Type IV patients were more complex. For type IVA, the unilateral axis pedicle and lateral mass (contralateral or ipsilateral) were all hypoplasia, pedicle screw could be performed on unilateral normal side, the other side could choose lateral mass screw or without screw; for type IVB, unilateral pedicle and bilateral lateral masses were dysplasia, we could choose unilateral pedicle screw to place in normal side and the other side without screw; for type IVC, bilateral pedicles and unilateral lateral mass were dysplasia, unilateral lateral mass screw should be placed in normal side and the other side without screw; for type IVD, bilateral pedicles and bilateral lateral masses were dysplasia, axis cross lamina screw was a good choice or axis without screw.

#### The Repeatability and Credibility of the Classification System Are Good

The classification of axis deformity in patients with BI is of great practical value. The repeatability and credibility of each type were good (Appendix), so this classification is reliable and feasible. There are many variations in the axis and great individual differences, which have a great influence on the axis screw placement. Our study clearly reveals that the most common axis deformities are pedicle and lateral mass, these variations can affect the placement of axis screws, especially the placement of pedicle screws. These variations can also increase the risk of vertebral artery injury and penetration of pedicle medial wall in the process of screw placement. According to the classification, we can know how to place the axis screw safely, each type has a guiding significance for axis screw placement, and the risks of axis screw misplacement are effectively avoided and the operation risks could be reduced.

### Study Limitations

This classification is summed up from only 92 cases of BI patients. This means that the sample size is relatively small and some rare axis variations have not have been found. It is necessary for more clinical cases to find new axis variations to continue to improve this classification. Moreover, this classification scheme needs to be continuously verified in a large number of clinical cases to test its practicability, so as to better improve the classification and provide guidance for the axis screw placement scheme in the BI and other upper cervical surgery.

### Conclusion

In our study, we found the incidence of axis malformation in patients with BI was as high as 42.4%, the axis deformities were divided into four types, and the most common malformation was type IV. Moreover, the repeatability and credibility of this classification scheme were very good, it could provide personalized guidance for the axis screw placement scheme in the BI and other upper cervical surgeries.

## Author Contributions

All authors had full access to the data in the study and take responsibility for the integrity of the data and the accuracy of the data analysis. Conceptualization, Q.T.H. and L.C.; Methodology, Q.T.H. and J.K.C.; Investigation, Q.T.H., J.K.C., H.C.T., B.C. and S.Y.W.; Formal Analysis, X.C.F., Y.P.Z. and J.L.W.; Writing – Original Draft, Q.T.H. and J.K.C.; Writing – Review & Editing, Y.P.Z. and J.L.W.; Supervision, L.N. and X.P.; Funding Acquisition, L.C.

## Conflict of Interest

The authors declare that they have no conflict of interest.

## Ethics Statement

The trial was conducted in accordance with the Declaration of Helsinki (as revised in 2013). The study was approved by the Ethics Committee of Qilu Hospital of Shandong University (NO. 2019264) and informed consent was taken from all individual participants who accepted surgery.

## Appendix

The repeatability analysis of type I

**Table:**

Researcher	The consistency of twice classifications (%)	Kappa value
1	85.6	0.851
2	87.2	0.889
3	83.4	0.836
4	84.6	0.825
Mean	85.2	0.85

The repeatability analysis of type II

**Table:**

Researcher	The consistency of twice classifications (%)	Kappa value
1	82.1	0.828
2	81.6	0.842
3	84.2	0.825
4	81.8	0.822
Mean	82.425	0.829

The repeatability analysis of type III

**Table:**

Researcher	The consistency of twice classifications (%)	Kappa value
1	83.2	0.854
2	84.8	0.862
3	85.1	0.837
4	83.5	0.844
Mean	84.15	0.849

The repeatability analysis of type IV

**Table:**

Researcher	The consistency of twice classifications (%)	Kappa value
1	82.2	0.833
2	79.8	0.823
3	80.1	0.831
4	79.9	0.824
Mean	80.5	0.828

The credibility analysis of type I

**Table:**

Researcher	The consistency of inter‐observer (%)	Kappa value
1–2	84.9	0.848
1–3	80.3	0.878
1–4	83.5	0.838
2–3	80.8	0.819
2–4	82.4	0.825
3–4	81.6	0.834
Mean	82.25	0.84

The credibility analysis of type II

**Table:**

Researcher	The consistency of inter‐observer (%)	Kappa value
1–2	77.9	0.817
1–3	78.5	0.808
1–4	81.4	0.828
2–3	76.4	0.809
2–4	79.8	0.819
3–4	82.6	0.846
Mean	79.43	0.821

The credibility analysis of type III

**Table:**

Researcher	The consistency of inter‐observer (%)	Kappa value
1–2	80.2	0.811
1–3	76.4	0.802
1–4	79.1	0.810
2–3	78.6	0.799
2–4	79.3	0.801
3–4	80.3	0.813
Mean	80.1	0.806

The credibility analysis of type IV

**Table:**

Researcher	The consistency of inter‐observer (%)	Kappa value
1–2	78.2	0.823
1–3	77.6	0.811
1–4	80.3	0.826
2–3	77.4	0.804
2–4	76.4	0.790
3–4	77.5	0.809
Mean	77.9	0.811
